# Plasma Metabolic and Inflammatory Protein Signatures in Psychiatric Disorders

**DOI:** 10.3390/ijms26136260

**Published:** 2025-06-28

**Authors:** Manel Naifar, Franklin Ducatez, Wassim Guidara, Manel Maalej, Celine Lesueur, Carine Pilon, Thomas Plichet, Mohamed Maalej, Fatma Ayadi, Soumeya Bekri

**Affiliations:** 1 Biochemistry Laboratory “Molecular Basis of Human Diseases”, LR19ES13, Sfax Medicine College, University of Sfax, Sfax 3029, Tunisia; dr.manelnaifar@gmail.com (M.N.); wassimguidara44@gmail.com (W.G.); mmanel84@yahoo.fr (M.M.); ayadi_fatma@medecinesfax.org (F.A.); 2 UNIROUEN, AIMS, CHU Rouen, Reference Center for Lysosomal Diseases, Filière Maladies Héréditaires du Métabolisme (G2M), Department of Metabolic Biochemistry, Normandie University, F-76000 Rouen, France; franklin.ducatez@chu-rouen.fr (F.D.); celine.lesueur@chu-rouen.fr (C.L.); carine.pilon@chu-rouen.fr (C.P.); thomas.plichet@chu-rouen.fr (T.P.); 3 Psychiatry C-Department, Hedi Chaker University Hospital, University of Sfax, Sfax 3029, Tunisia; mohamedmaalej81@gmail.com

**Keywords:** psychiatric disorders, schizophrenia, bipolar disorder, schizoaffective disorder, omics, proteomics, metabolomics

## Abstract

Psychiatric disorders, particularly schizophrenia (SCZ), bipolar disorder (BD), and schizoaffective disorder (SAD), present significant diagnostic challenges. Current diagnostic methods rely on clinical observation and self-reported symptoms, leading to under-diagnosis and delayed treatment. To address this gap, we applied mass spectrometry-based metabolomic profiling and targeted analysis of inflammatory proteins to plasma samples from patients versus controls, aiming to uncover disease-related molecular patterns and enhance our understanding of the underlying pathophysiology of these complex disorders. This study included 26 patients with BD, 34 with SCZ, 16 with SAD, and age- and sex-matched controls. All diagnoses were established according to DSM-5 criteria. Unsupervised analysis shows a clear separation between controls and patients, indicating distinct metabolic and inflammatory profiles. However, the lack of clear differentiation among the three disease subgroups suggests shared biological profiles across these psychiatric disorders. Biomolecules driving this separation between controls and patients includes decreased levels of proinflammatory cytokines, amino acids, and glycerophospholipids, and increased levels of acylcarnitines. This study represents a step towards addressing the limitations of current diagnostic approaches to severe psychiatric disorders, which rely heavily on clinical symptoms, by using omics approaches to refine their diagnosis and treatment.

## 1. Introduction

Psychiatric disorders are complex and often under-/misdiagnosed, with a heavy socioeconomic impact. In particular, schizophrenia (SCZ), bipolar disorder (BD), and schizoaffective disorder (SAD) are among the most severe mental illnesses, with an estimated prevalence of 3% of the general population for all psychotic disorders [1] and 2.4% for bipolar disorder [2]. Unfortunately, despite their high prevalence, the pathophysiology of these complex diseases is still unclear. Thus, tools for diagnosis and risk prediction are limited. In clinical practice, the diagnosis of those disorders is based on self-reported symptoms (or those observed by family and friends) and/or those observed by clinicians, with reference to the diagnostic criteria of the *Diagnostic and Statistical Manual of Mental Disorders* of the American Psychiatric Association (DSM-5) [3]. The other primary system for psychiatric disorder classification is the International Classification of Diseases (ICD-11) [4]. Both DSM-5 and ICD-11 face significant criticism. The DSM-5 has been questioned for its risk of overdiagnosis and poor reliability [5], while the ICD-11 includes broad symptom definitions that may reduce diagnostic precision and cultural bias [6]. Additionally, there is considerable symptom overlap between psychiatric and neurological conditions, hampering diagnoses and highlighting the need for additional tools to refine the diagnosis in the psychiatry field [7,8,9].

The emerging Research Domain Criteria (RDoC) initiative seeks to integrate multiple levels of information and focus on common processes across disorders [10]. These classification systems evolve and integrate the growing understanding of mental health and the complex interplay of biological, psychological, environmental, and social factors in psychiatric disorders.

Psychiatric disorders are known to have substantial heritability, with numerous susceptibility genes involved in their underlying pathophysiology. Genome-wide association studies (GWAS) have identified common risk variants affecting neurodevelopment, synaptic transmission, and immune function [11,12]. Alterations in the methylenetetrahydrofolate reductase (*MTHFR*) gene have been linked to disruptions in folate metabolism, DNA methylation processes, and neurotransmitter synthesis, contributing to metabolic imbalances observed in psychiatric patients [13,14].

Therefore, the identification of biomarkers for these disorders may significantly improve patient stratification, enable earlier intervention, and refine personalized treatment strategies. Omics sciences represent a powerful, holistic approach, enabling high-throughput analysis of different classes of biomolecules within a sample. This comprehensive approach can capture the molecular complexity underlying these conditions and facilitates understanding of disease mechanisms, stratifying patients, and developing targeted therapies [15]. In particular, proteomics and metabolomics are omics technologies with high potential to parse complex biological processes. Being close to the phenotype, analyzing metabolites and proteins within body fluids allows for interrogating metabolic shifts that reflect genetic variation, physiological changes, and disease. In this study, we used plasma-targeted metabolomic and proteomic approaches in patients with SCZ, SAD, and BD. Our objective was to explore disease-related omics patterns that may provide insights into the pathophysiology of these conditions and to identify biological markers capable of distinguishing patients with psychiatric disorders from healthy controls.

## 2. Results

Our patient cohort was composed of individuals diagnosed with schizoaffective disorder (n = 16), bipolar disorder (n = 26), and schizophrenia (n = 34). The cohort is mostly homogeneous, aged around 34 years old, with no clear higher prevalence of obesity. The majority of the cohort was urban, living with family, with an educational level equal to or above secondary, and declaring tobacco or psychoactive substance use. There was a higher prevalence of married patients in the bipolar subgroup (36%, adj-p.val = 0.016) and having an active profession (60%, adj-p.val = 0.016).

The metabolomic and proteomic profiling data (Supplementary Table S2) of the control and patient groups were analyzed using an unsupervised approach, principal component analysis (PCA), along with a differential expression analysis to assess metabolic shifts and inflammatory marker variations associated with patient conditions.

**Figure 1:**
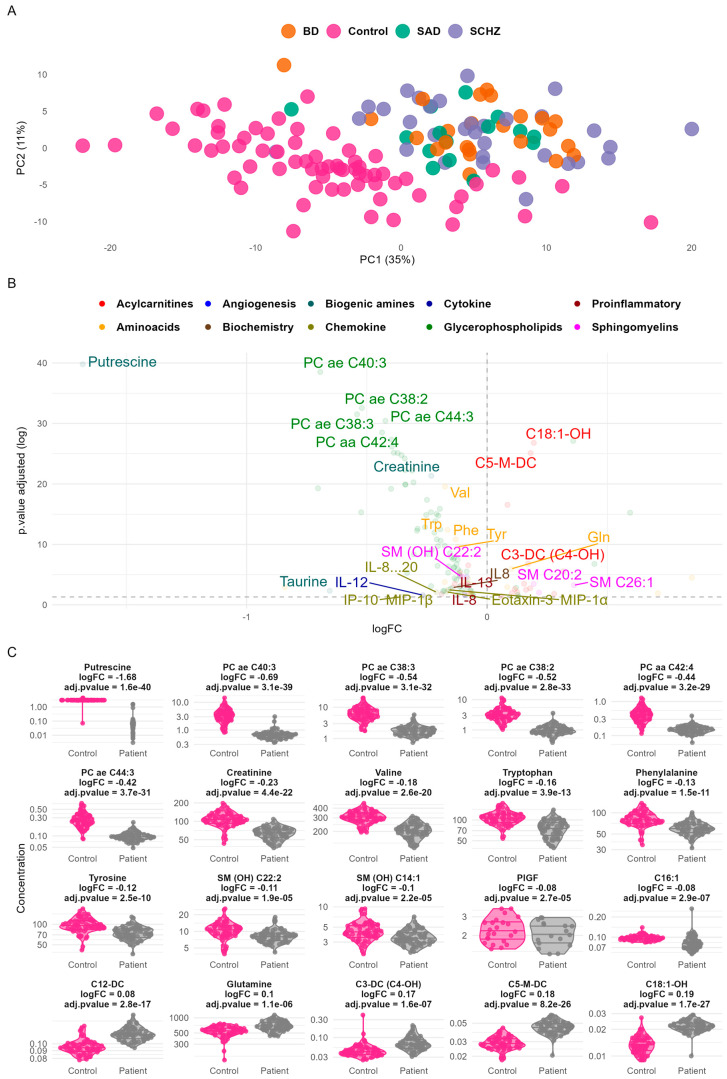
Principal component analysis (PCA) and differential expression analysis of metabolic and inflammatory markers in control vs. patient groups. (**A**) PCA plot highlighting a clear separation between control and patient samples based on PC1 (35%) and PC2 (11%). Samples are color-coded by group: Control (black), schizophrenia—SCZ (green), bipolar disorder—BD (blue), and schizoaffective disorder—SAD (red). The top metabolites contributing to group separation are annotated. (**B**) A volcano plot of differential expression analysis, showing log-fold changes (logFC) and adjusted *p*-values of metabolites and cytokines, categorized into functional groups (e.g., acylcarnitines, biogenic amines, and glycerophospholipids). Significant markers are labeled, with the key upregulated and downregulated metabolites highlighted. (**C**) Violin plot of the top differentially expressed features.

In Figure 1A, the PCA score plot highlights a group separation along the principal components PC1 (35% variance explained) and PC2 (11% variance explained), suggesting distinct underlying metabolic and inflammatory profiles between the control and patient groups. The lack of clear separation among the three disease subgroups suggests the existence of shared biological profiles across these distinct psychiatric disorders. 

The main biomolecules contributing to this separation between controls and patients are acylcarnitines, amino acids, biogenic amines, glycerophospholipids, and proinflammatory cytokines (Figure 1B,C, and Supplementary Tables S3–S5). The most differentially expressed biomolecules between the two groups were metabolites like putrescine, glycerophospholipids (PC ae C40:3, PC ae C38:2, PC ae C44:3, PC ae C38:3, and PC aa C42:4), creatinine, acylcarnitines (C18:1-OH, C5-M-DC, and C3-DC), sphingomyelines (SM (OH) C22:2, SM C26:1, and SM C20:2), and amino acids (valine, tryptophan, phenylalanine, tyrosine, glutamine, and taurine). Key inflammatory markers such as IL-8, IL-12, and IL-13 were downregulated in patients.

**Figure 2:**
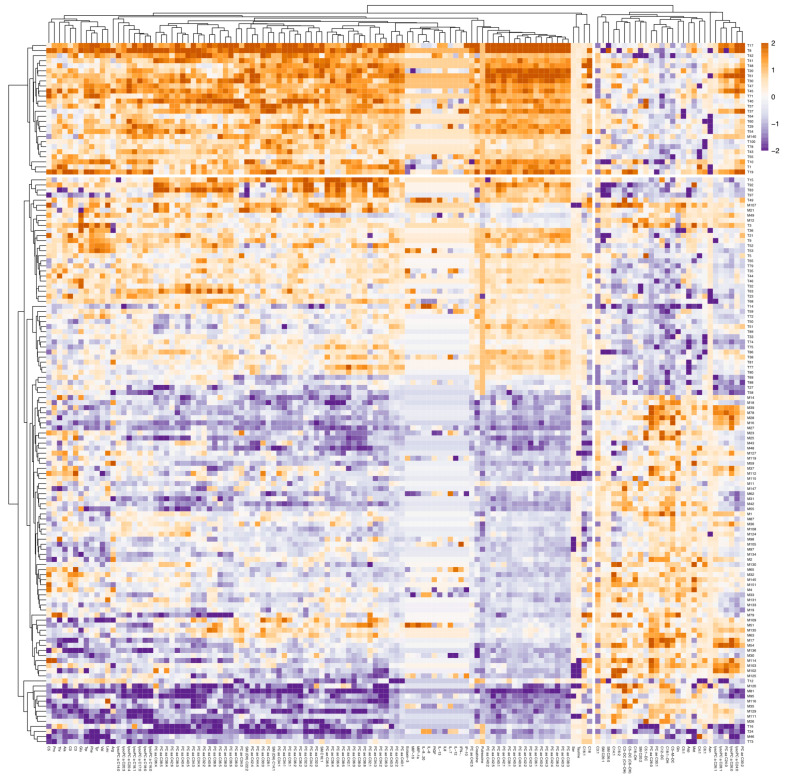
Heatmap of top differentially expressed features between patients and controls. Rows represent samples, with M denoting patient samples and T denoting control samples. Columns represent metabolic and inflammatory markers, organized by class (amino acids, lysophosphatidylcholines, phosphatidylcholines, sphingomyelins, and cytokines). Color intensity corresponds to normalized expression levels, with red indicating higher expression and blue indicating lower expression.

To visualize the expression patterns of the different features, we used a heatmap, as shown in Figure 2, highlighting top metabolites and proteins that distinguish the patient and control groups. The patient samples exhibit unique metabolic and inflammatory profiles. Patients have lower levels of amino acids, such as valine, tryptophan, phenylalanine, tyrosine, and taurine, while glutamine levels are high.

As shown in Table 1, there were significant differences in lipid profiles between patients and controls, with lower levels of total cholesterol, LDL-C, triglycerides (TG), phosphatidylcholines (PC), lysophosphatidylcholines (LPC), sphingomyelins (SM), and acylcarnitines observed in the patient group.

In terms of inflammatory cytokines, IL-8, MIP-1, IP-10, IL-13, and Eotaxin-3 showed lower levels in patient samples compared to the controls.

**Figure 3:**
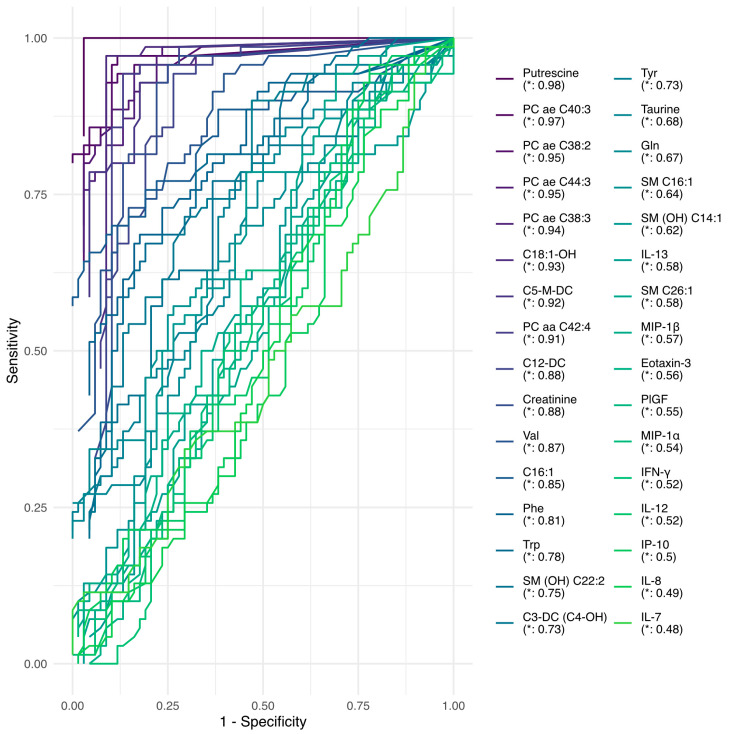
The receiver operating characteristic (ROC) curves of decision tree models. The area under the curve (AUC) indicates model predictive performance.

Furthermore, we explored the predictive performance of each of the features using predictive decision tree models. The area under the curve and receiver operating characteristic curves were used as performance metrics. All model-related results are presented in Supplementary Table S6. The top models are shown in Figure 3, which highlights the following: Putrescine (AUC = 0.98), PC ae C40:3 (AUC = 0.96), PC ae C38:2 (AUC = 0.96), PC ae C44:3 (AUC = 0.95), C5-M-DC (AUC = 0.94), PC ae C38:3 (AUC = 0.94), C18:1-OH (AUC = 0.93), PC aa C42:4 (AUC = 0.93), C12-DC (AUC = 0.88), valine (AUC = 0.87), creatinine (AUC = 0.87), C16:1 (AUC = 0.85), phenylalanine (AUC = 0.8), tryptophan (AUC = 0.75), SM (OH) C22:2 (AUC = 0.75), tyrosine (AUC = 0.73), C3-DC (C4-OH) (AUC = 0.72), taurine (AUC = 0.69), glutamine (AUC = 0.68), SM C20:2 (AUC = 0.68), IL8 (AUC = 0.64), and SM C16:1 (AUC = 0.63).

## 3. Discussion

This study investigated the metabolic and inflammatory profiles of patients with psychiatric disorders compared to healthy controls. The study included three subgroups of patients: schizophrenia, bipolar disorder, and schizoaffective disorder. An omics approach, combining metabolomics and proteomics analysis targeting inflammatory markers, was applied. The PCA score plot (Figure 1A) and heatmap (Figure 2) show a clear separation between patient and control groups. This separation indicates distinct metabolic and inflammatory profiles between patients with psychiatric disorders and healthy controls. Interestingly, the three patient subgroups did not show clear differentiation, suggesting shared biological profiles across these distinct psychiatric disorders. This finding reinforces the growing recognition of transdiagnostic processes in psychiatry and challenges the traditional categorical description of mental disorders [16,17]. Interestingly, the biological profile driving this separation is organized around two clusters: one containing mostly phosphatidylcholines and amino acids whose levels are reduced compared to the control group, and one displaying increased acyl-carnitine levels. However, the analyzed panel in this study may not include markers specific to SCZ, BP, or SAD. Broader panels of metabolites and proteins could improve the stratification of psychiatric disorders and reveal disorder-specific biological mechanisms.

The results revealed significant alterations in the levels of several amino acids crucial to neurotransmitter metabolism. Decreased concentrations of valine, tryptophan, phenylalanine, tyrosine, and taurine were observed. In contrast, glutamine levels were found to be elevated. The link between low tryptophan levels and depression has been reported in numerous studies [18,19,20]. Tryptophan, an essential amino acid, is a precursor of serotonin and kynurenine. Serotonin is a neurotransmitter involved in mood regulation, cognition, appetite, and sleep regulation. Low levels of serotonin have been associated with mood disorders, including depression and anxiety. This connection led to the development of selective serotonin reuptake inhibitors (SSRIs) as a treatment for depression. The kynurenine pathway has been heavily incriminated in depressive states [21]. Dietary intake of tryptophan and gut microbiota balance may modulate the amount of tryptophan reaching the brain. The “gut-brain axis” is an area of active research, particularly for its involvement in mental disorders and its therapeutic potential [22,23,24,25,26,27,28,29]. Tyrosine and phenylalanine are precursors for dopamine and norepinephrine. Their depletion may affect mood, cognition, and behavior [30].

Importantly, a recent metabolomics study conducted on adolescents diagnosed with major depressive disorder, bipolar disorder, or SCZ revealed similar metabolic patterns, with decreased concentrations of tryptophan, tyrosine, and phenylalanine in the affected adolescents [31]. Valine, a branched-chain amino acid (BCAA), plays a significant role in brain function and protein synthesis. Its reduction could indeed affect brain metabolism by modulating neurotransmitter balance [32]. Interestingly, glutamine levels were elevated in patients versus control individuals. Glutamate–glutamine is crucial for excitatory neurotransmission in the brain. Its disruption has been reported in several psychiatric disorders, including SCZ and mood disorders [33,34,35,36].

Of note, the most discriminative metabolic feature in our study was putrescine, which displayed lower levels in patients vs. controls. Putrescine is a polyamine involved in numerous cell processes such as proliferation, neuroinflammation, and oxidative stress. Polyamine levels in the brain seem to be precisely regulated. Lower levels are associated with abnormal brain development or increased vulnerability to neurodevelopmental disorders [37]. On the contrary, higher levels of polyamines have been reported in numerous neuropsychiatric conditions and may contribute to glutamatergic dysregulation, neuronal excitotoxicity, and blood–brain barrier permeability changes seen in SCZ and mood disorders [37]. Accordingly, a recent multi-omics analysis in a SCZ model based on patient-derived induced pluripotent stem cells (iPSCs) revealed significant alteration of polyamine and gamma-aminobutyric acid (GABA) metabolism with downregulation of the glutamate decarboxylase encoding genes *GAD1* and *GAD2* [38].

Significant changes in various lipid categories, including lysophosphatidylcholines, phosphatidylcholines, sphingomyelins, and acylcarnitines, have been observed. Phospholipid alterations could have several consequences, such as (i) cell membrane disruption, potentially affecting neuronal function and synaptic transmission, (ii) alterations in lipid-based signaling pathways involved in neurotransmission and neuroplasticity, and (iii) myelination disturbances, as sphingomyelins are important components of myelin sheaths. Importantly, alterations in membrane phospholipid composition have been documented in SCZ and BP [39,40,41]. Disturbances in phospholipid metabolism can alter neuronal membrane structure, which in turn affects neurotransmitter systems and ion channel function. Accordingly, recent lipidomic studies indicate that these membrane lipid abnormalities may play a role in disrupting dopamine signaling, potentially influencing symptom severity and cognitive function in SCZ and BD [42,43]. A recent study suggests that changes in glycerophospholipid metabolism could act as a link between gut microbiota and depression [44].

Moreover, changes in acylcarnitine levels have been observed, suggesting potential disruptions in energy metabolism, which aligns with growing evidence of mitochondrial dysfunction in psychiatric disorders [45].

In contrast with the common inflammatory hypothesis in psychiatric disorders [46], this study found downregulation of several pro-inflammatory cytokines in patients, including IL-8, IL-12, and IL-13. Additionally, lower levels of MIP-1, IP-10, and Eotaxin-3 were observed in patient samples compared to controls. This unexpected finding needs to be further investigated and may suggest a compensatory anti-inflammatory response to chronic low-grade inflammation.

The observed variations between patient and control groups may be partially driven by underlying genetic factors, such as *MTHFR* variants [13,14]. Moreover, behavioral factors such as diet, physical activity, substance use (e.g., smoking and alcohol consumption), and medication effects may further modulate metabolic and inflammatory profiles. These influences highlight the complexity of disentangling disease-specific molecular signatures from secondary or lifestyle-related effects.

This study has several limitations. First, while plasma-based metabolomic and proteomic analyses provide accessible biomarkers, they may not fully capture the biochemical complexity of the central nervous system (CNS). Second, the relatively small sample size within each diagnostic group may limit the statistical power for detecting subtle subgroup differences. Finally, although targeted panels offer high sensitivity for selected analytes, untargeted approaches could uncover additional, potentially relevant biomarkers. Future studies with larger cohorts and expanded molecular coverage will be necessary to validate and extend these findings.

## 4. Materials and Methods

### 4.1. Participants

A total of 26 patients with BD, 34 patients with SCZ, and 16 patients with SAD were included in this study. The SCZ and BD diagnoses were established according to the DSM-5 [3]. All enrolled patients were symptomatic and drug-free for at least three months before hospitalization. Age-matched, healthy control volunteers from the same region were recruited. Only patients of male sex/gender were enrolled because this psychiatry department hosts only male patients.

SCZ patients were assessed using the Positive and Negative Symptoms Scale (PANSS) [47]. Depressive and manic symptoms were assessed for BD patients using the Montgomery–Asberg Depression Rating Scale (MADRS) [48]. For the severity of mania, the Bech and Rafaelsen scale [49] and Montreal Cognitive Assessment (MoCA) [50] were applied for all patients. Patients with a history of dementia or other psychiatric or neurological disorders were excluded. Eighty control individuals were included in the study. Exclusion criteria for the control subjects included personal or family history of psychiatric disorders. Blood samples were collected in EDTA-coated tubes for plasma metabolite determination after 8 h of fasting. Samples were centrifuged at 20 °C and 1800 g for 15 min and stored at −80 °C until analysis.

The study was approved by the Local Ethics Committee of Hedi Chaker Hospital (CPP SUD N°351/2021) and performed in accordance with the Declaration of Helsinki. All subjects provided written informed consent prior to inclusion in the study. The full cohort is described in Table 2 and Supplementary Table S1.

### 4.2. Targeted Metabolomics Analysis

Sample preparation was carried out according to the manufacturer’s protocol. Briefly, 10 µL of plasma was transferred to the upper 96-well plate and dried under a nitrogen stream. Thereafter, 50 µL of a 5% phenylisothiocyanate solution was added to derivatize amino acids and biogenic amines. After incubation, the spots were dried again before the metabolites were extracted using 5 mM ammonium acetate in methanol (300 µL) into the lower 96-well plate for analysis after further dilution using the MS running solvent A. Quantification was carried out according to the manufacturer’s protocol using isotopically labeled internal standards and a calibration curve [51]. Endogenous metabolites were analyzed using the AbsoluteIDQ^®^ p180 Kit (BIOCRATES Life Science AG, Innsbruck, Austria) through a targeted, quantitative, and quality-controlled assay. The workflow involved flow injection analysis (FIA) and HPLC methods followed by mass spectrometry on an API 4000 QTrap (Sciex, USA).

The analysis was performed on a triple-quadrupole mass spectrometer coupled with a liquid chromatography system (Shimadzu LC-20AB with autosampler SIL-20AC, Prominence, Kyoto, Japan). Samples were processed according to the manufacturer’s protocol, with key parameters including an autosampler temperature of 10 °C, an injection volume of 10 µL, and a reversed-phase HPLC gradient using HPLC-grade water and acetonitrile, both containing 0.2% formic acid, at a flow rate of 0.5 mL/min. For FIA, acetonitrile with 0.2% formic acid was used at a maximum flow rate of 0.2 mL/min. [51]. The full list of the 188 measured metabolites is presented in Supplementary Table S7: 21 amino acids, 21 biogenic amines, 1 monosaccharide, 40 acylcarnitines, 90 glycerophospholipids, and 15 sphingolipids.

The determination of amino acids and biogenic amines was performed using reverse-phase liquid chromatography coupled to a mass spectrometer in multiple reaction monitoring (MRM) mode. The determination of lipids and hexoses was carried out through direct injection via FIA-MRM in positive and negative ionization modes. Data acquisition and processing were performed using Analyst 1.5 software (Sciex, Framingham, MA, USA).

### 4.3. Targeted Proteomics Analysis

Plasma proteins were measured using the V-PLEX Human biomarker 40-Plex kit (Meso Scale Discovery, MA, USA). The list is presented in Supplementary Table S7. Inflammatory biomarkers were assayed using various V-PLEX panels such as cytokines, chemokines, and angiogenesis and vascular panels including C-reactive protein (CRP), interferon gamma (IFN-γ), interleukin 1α, interleukin 1β, interleukin 2, interleukin 4, interleukin 5, interleukin 6, interleukin 7, interleukin 8, IP-10, interleukin 10, interleukin 12/interleukin 23p40, interleukin 12p70, interleukin 13, interleukin 15, interleukin 16, interleukin 17A, Eotaxin, Eotaxin-3, fibroblast growth factor 2 (FGF2), granulocyte–macrophage colony-stimulating factor (GM-CSF), intercellular adhesion molecule 1 (ICAM-1), Monocyte chemoattractant protein 1 (MCP-1), Monocyte chemoattractant protein 4 (MCP-4), macrophage-derived chemokine (MDC), macrophage inflammatory proteins 1 alpha (MIP-1α), macrophage inflammatory proteins 1 beta (MIP-1β), placental growth factor (PlGF), Serum amyloid A (SAA), thymus- and activation-regulated chemokine (TARC), Angiopoietin-1 receptor (Tie-2), tumor necrosis factor alpha (TNF-α), tumor necrosis factor beta (TNF-β), vascular cell adhesion protein 1 (VCAM-1), vascular endothelial growth factor (VEGF-A), vascular endothelial growth factor C (VEGF-C), vascular endothelial growth factor D (VEGF-D), and vascular endothelial growth factor receptor 1 (VEGFR-1/Flt-1). Data were acquired using a SECTOR S 6000 plate reader (Meso Scale Diagnostics, Rockville, MD, USA) [52].

### 4.4. Data Analysis

All data analyses and visualizations were performed using R software (4.2.1) [53]. Clinical variables were compared between disease subgroups using the Chi2 test or Fisher’s exact test and the Kruskal–Wallis test. General biochemistry lipidic profiling was compared between controls and patients using Student’s *t*-test.

Prior to downstream analysis, metabolite and protein levels were log-transformed and Pareto-scaled [54]; missing values were imputed using the nearest neighbor averaging algorithm using the impute.knn function in the impute R package.

Unsupervised exploration of the biological profiles was performed with Principal Component Analysis. Correlation analysis was performed using the Spearman correlation.

Differential analysis was performed using the Limma package [55], with sex and age taken into account as cofounders. Differently expressed biomolecules were then clustered using the Euclidean distance and visualized within a heatmap. False discovery rates were corrected using the Benjamini–Hochberg–Yekutieli method [56], and adjusted *p* < 0.05 was considered statistically significant.

The discriminatory potential of each differentially expressed feature between controls and patients was evaluated using decision trees with the ranger package [57] and the caret package in R [58]. Each decision tree model was built using one biomolecule at a time. Then, the discriminative performances of each model were assessed with the MLeval package in R using the area under the curve (AUC) for the resulting receiver operating characteristic (ROC) curve.

## 5. Conclusions

In conclusion, the lack of clear differentiation among the three patient subgroups suggests shared biological underpinnings across different psychiatric disorders. This finding supports the need for transdiagnostic approaches in psychiatric research and treatment. The identified metabolites and inflammatory markers could serve as potential biomarkers for psychiatric disorders. Biomarkers may offer a robust diagnostic or prognostic tool, pending validation of specificity and sensitivity in larger cohorts. Moreover, longitudinal studies to track how these profiles evolve with or without treatment may reveal the causal relationships between biological alterations and psychiatric symptoms.

This study provides insights into the complex metabolic and inflammatory landscape associated with psychiatric disorders. The results underline the need for integrated approaches to better understand the mechanisms underlying mental disorders, to develop a biomarker-based diagnostic strategy, and to identify targeted treatments in psychiatry.

## Figures and Tables

**Table 1:** Lipid concentrations in the studied patients compared to controls.

Total Population Lipids		Controls			Patients		*p*-Value
	n	Mean	SD	n	Mean	SD	
**TC (mmol/L)**	68	4.41	0.87	70	3.64	0.77	**1.57 × 10^−7^**
**TG (mmol/L)**	68	1.17	0.50	70	1.01	0.43	**3.85 × 10^−2^**
**HDL-C (mmol/L)**	39	1.08	0.25	48	1.18	0.43	1.76 × 10^−1^
**LDL-C (mmol/L)**	39	2.72	0.75	48	2.08	0.71	**1.11 × 10^−4^**
Tota PC (µmol/L) *	68	1084.83	283.71	70	774.33	156.28	**2.65 × 10^−12^**
Total LPC (µmol/L) *	68	410.50	152.54	70	252.88	88.92	**3.41 × 10^−11^**
Total SM (µmol/L) *	67	255.81	75.75	70	228.15	52.16	**1.46 × 10^−2^**
Total Acylcarnitines (µmol/L) *	68	56.04	15.87	70	48.18	12.77	**1.74 × 10^−3^**

TC: Total cholesterol, TG: triglycerides, HDL-C: high-density lipoprotein cholesterol, LDL-C: low-density lipoprotein cholesterol, PC: phosphatidylcholines, LPC: lysophosphatidylcholines, SM: sphingomyeline; *p* significative if <0.05. *: It is the sum of all assessed species in each class (Supplementary Table S2).

**Table 2:** Cohort overview.

Characteristic	Schizoaffective Disorder (SAD), N = 16 ^1^	Bipolar Disorder (BD), N = 26 ^1^	Schizophrenia (SCZ), N = 34 ^1^	*p*-Value ^2^	q-Value ^3^
Age	30 (24, 33)	36 (29, 47)	36 (29, 44)	0.022	0.070
Unknown	0	1	0		
Number of Tobacco Packs/Year	0 (0, 4)	2 (0, 12)	0 (0, 15)	0.7	0.7
Unknown	2	7	6		
Weight	60 (57, 80)	64 (60, 71)	64 (57, 70)	0.7	0.7
Unknown	3	8	7		
Height	1.72 (1.70, 1.78)	1.75 (1.66, 1.79)	1.70 (1.66, 1.75)	0.5	0.7
Unknown	4	11	9		
Body Mass Index (BMI)	20.5 (18.8, 26.2)	21.8 (20.9, 23.3)	21.7 (19.3, 25.6)	0.9	0.9
Unknown	4	11	9		
Marital Status				0.002	**0.016**
Divorced	0 (0%)	1 (4.0%)	0 (0%)		
Married	0 (0%)	9 (36%)	3 (8.8%)		
Unmarried	16 (100%)	15 (60%)	31 (91%)		
Unknown	0	1	0		
Living Situation				0.4	0.6
Alone	2 (13%)	2 (8.0%)	7 (21%)		
With family	13 (87%)	23 (92%)	27 (79%)		
Unknown	1	1	0		
Educational Level				0.2	0.5
Illiterate	2 (12%)	2 (8.0%)	6 (18%)		
Primary	5 (31%)	4 (16%)	13 (38%)		
Secondary	6 (38%)	11 (44%)	12 (35%)		
University-level	3 (19%)	8 (32%)	3 (8.8%)		
Unknown	0	1	0		
Social Level				0.017	0.070
High	0 (0%)	2 (9.1%)	0 (0%)		
Low	10 (67%)	10 (45%)	24 (86%)		
Medium	5 (33%)	10 (45%)	4 (14%)		
Unknown	1	4	6		
Profession				0.003	**0.016**
Active	4 (27%)	15 (60%)	6 (18%)		
Inactive	11 (73%)	10 (40%)	28 (82%)		
Unknown	1	1	0		
Housing				0.3	0.6
Rural	4 (29%)	8 (33%)	15 (48%)		
Urban	10 (71%)	16 (67%)	16 (52%)		
Unknown	2	2	3		
Psychoactive Substance	8 (50%)	20 (83%)	19 (56%)	0.045	0.12
Unknown	0	2	0		
Tobacco Use	8 (50%)	19 (76%)	17 (50%)	0.10	0.2
Unknown	0	1	0		

^1^ Median (IQR); n (%). ^2^ Kruskal–Wallis rank-sum test; Fisher’s exact test; Pearson’s Chi-squared test. ^3^ False discovery rate correction for multiple testing.

## Data Availability

The original contributions presented in this study are included in the article/Supplementary Materials. Further inquiries can be directed to the corresponding author.

## Supplementary Materials

The supporting information can be downloaded at https://www.mdpi.com/article/10.3390/ijms26136260/s1.

## Abbreviations

The following abbreviations are used in this manuscript:

AUC	Area Under the Curve
BD	Bipolar Disorder
DSM-5	*Diagnostic and Statistical Manual of Mental Disorders, 5th Edition*
EDTA	Ethylenediaminetetraacetic Acid
FGF2	Fibroblast Growth Factor 2
FIA	Fluorescence Immunoassay
HPLC	High-Performance Liquid Chromatography
ICAM-1	Intercellular Adhesion Molecule 1
ICD-11	International Classification of Diseases, 11th Revision
LDL	Low-Density Lipoprotein
LPC	Lysophosphatidylcholine
MADRS	Montgomery–Åsberg Depression Rating Scale
MoCA	Montreal Cognitive Assessment
MRM	Multiple Reaction Monitoring
PANSS	The Positive and Negative Symptoms Scale
PC	Phosphatidylcholine
PLGF	Placental Growth Factor
RDoC	Research Domain Criteria
SAA	Serum Amyloid A
SAD	Schizoaffective Disorder
SCZ	Schizophrenia
SM	Sphingomyelin
TARC	Thymus- and Activation-Regulated Chemokine
TC	Total cholesterol
TG	Triglycerides
Tie-2	Tyrosine Kinase with Immunoglobulin-Like and EGF-Like Domains 2
TNF-β	Tumor necrosis factor beta
VCAM-1	Vascular Cell Adhesion Molecule 1
VEGF	Vascular Endothelial Growth Factor
VEGFR	Vascular Endothelial Growth Factor Receptor

## References

[1] Perälä J., Suvisaari J., Saarni S.I., Kuoppasalmi K., Isometsä E., Pirkola S., Partonen T., Tuulio-Henriksson A., Hintikka J., Kieseppä T. (2007). Lifetime prevalence of psychotic and bipolar I disorders in a general population. Arch. Gen. Psychiatry.

[2] Zhong Y., Chen Y., Su X., Wang M., Li Q., Shao Z., Sun L. (2024). Global, regional and national burdens of bipolar disorders in adolescents and young adults: A trend analysis from 1990 to 2019. Gen. Psychiatry.

[3] Tandon R., Gaebel W., Barch D.M., Bustillo J., Gur R.E., Heckers S., Malaspina D., Owen M.J., Schultz S., Tsuang M. (2013). Definition and description of schizophrenia in the DSM-5. Schizophr. Res..

[4] Reed G.M., First M.B., Kogan C.S., Hyman S.E., Gureje O., Gaebel W., Maj M., Stein D.J., Maercker A., Tyrer P. (2019). Innovations and changes in the ICD-11 classification of mental, behavioural and neurodevelopmental disorders. World Psychiatry.

[5] Wakefield J.C. (2015). DSM-5, psychiatric epidemiology and the false positives problem. Epidemiol. Psychiatr. Sci..

[6] Sharan P., Hans G. (2021). Cultural Issues Related to ICD-11 Mental, Behavioural and Neurodevelopmental Disorders. Consort. Psychiatr..

[7] Frances A.J., Nardo J.M. (2013). ICD-11 should not repeat the mistakes made by DSM-5. Br. J. Psychiatry.

[8] Kamp-Becker I. (2024). Autism spectrum disorder in ICD-11-a critical reflection of its possible impact on clinical practice and research. Mol. Psychiatry.

[9] Stein D.J., Lund C., Nesse R.M. (2013). Classification systems in psychiatry: Diagnosis and global mental health in the era of DSM-5 and ICD-11. Curr. Opin. Psychiatry.

[10] Insel T., Cuthbert B., Garvey M., Heinssen R., Pine D.S., Quinn K., Sanislow C., Wang P. (2010). Research domain criteria (RDoC): Toward a new classification framework for research on mental disorders. Am. J. Psychiatry.

[11] Musumeci A., Vinci M., Treccarichi S., Greco D., Rizzo B., Gloria A., Federico C., Saccone S., Musumeci S.A., Calì F. (2025). Potential Association of the CSMD1 Gene with Moderate Intellectual Disability, Anxiety Disorder, and Obsessive–Compulsive Personality Traits. Int. J. Mol. Sci..

[12] Ursini G., Di Carlo P., Mukherjee S., Chen Q., Han S., Kim J., Deyssenroth M., Marsit C.J., Chen J., Hao K. (2023). Prioritization of potential causative genes for schizophrenia in placenta. Nat. Commun..

[13] Hill M., Shannahan K., Jasinski S., Macklin E.A., Raeke L., Roffman J.L., Goff D.C. (2011). Folate supplementation in schizophrenia: A possible role for MTHFR genotype. Schizophr. Res..

[14] van Winkel R., Rutten B.P., Peerbooms O., Peuskens J., van Os J., De Hert M. (2010). MTHFR and risk of metabolic syndrome in patients with schizophrenia. Schizophr. Res..

[15] Tebani A., Afonso C., Marret S., Bekri S. (2016). Omics-Based Strategies in Precision Medicine: Toward a Paradigm Shift in Inborn Errors of Metabolism Investigations. Int. J. Mol. Sci..

[16] Dalgleish T., Black M., Johnston D., Bevan A. (2020). Transdiagnostic approaches to mental health problems: Current status and future directions. J. Consult. Clin. Psychol..

[17] Wise T., Robinson O.J., Gillan C.M. (2023). Identifying Transdiagnostic Mechanisms in Mental Health Using Computational Factor Modeling. Biol. Psychiatry.

[18] Comai S., Bertazzo A., Brughera M., Crotti S. (2020). Tryptophan in health and disease. Adv. Clin. Chem..

[19] Myint A.M., Kim Y.K., Verkerk R., Scharpe S., Steinbusch H., Leonard B. (2007). Kynurenine pathway in major depression: Evidence of impaired neuroprotection. J. Affect. Disord..

[20] Ogyu K., Kubo K., Noda Y., Iwata Y., Tsugawa S., Omura Y., Wada M., Tarumi R., Plitman E., Moriguchi S. (2018). Kynurenine pathway in depression: A systematic review and meta-analysis. Neurosci. Biobehav. Rev..

[21] Zong L., Ge M., Wang J., Kuang D., Wei H., Wang Z., Hu Z., Zhao C., Jin Q., Chen M. (2024). Causal association between kynurenine and depression investigated using two-sample mendelian randomization. Sci. Rep..

[22] Bogielski B., Michalczyk K., Głodek P., Tempka B., Gębski W., Stygar D. (2024). Association between small intestine bacterial overgrowth and psychiatric disorders. Front. Endocrinol..

[23] Collins S.M., Surette M., Bercik P. (2012). The interplay between the intestinal microbiota and the brain. Nat. Rev. Microbiol..

[24] MacKay M., Yang B.H., Dursun S.M., Baker G.B. (2024). The Gut-Brain Axis and the Microbiome in Anxiety Disorders, Post-Traumatic Stress Disorder and Obsessive-Compulsive Disorder. Curr. Neuropharmacol..

[25] Mhanna A., Martini N., Hmaydoosh G., Hamwi G., Jarjanazi M., Zaifah G., Kazzazo R., Haji Mohamad A., Alshehabi Z. (2024). The correlation between gut microbiota and both neurotransmitters and mental disorders: A narrative review. Medicine.

[26] Mulder D., Jakobi B., Shi Y., Mulders P., Kist J.D., Collard R.M., Vrijsen J.N., van Eijndhoven P., Tendolkar I., Bloemendaal M. (2024). Gut microbiota composition links to variation in functional domains across psychiatric disorders. Brain Behav. Immun..

[27] Wang Y., Kasper L.H. (2014). The role of microbiome in central nervous system disorders. Brain Behav. Immun..

[28] Xiao L., Liu S., Wu Y., Huang Y., Tao S., Liu Y., Tang Y., Xie M., Ma Q., Yin Y. (2023). The interactions between host genome and gut microbiome increase the risk of psychiatric disorders: Mendelian randomization and biological annotation. Brain Behav. Immun..

[29] Zhao G., Lu Z., Liao Y., Sun Y., Zhang Y., Kang Z., Feng X., Sun J., Yue W. (2025). Association of intestinal anti-inflammatory drug target genes with psychiatric Disorders: A Mendelian randomization study. J. Adv. Res..

[30] Roiser J.P., McLean A., Ogilvie A.D., Blackwell A.D., Bamber D.J., Goodyer I., Jones P.B., Sahakian B.J. (2005). The subjective and cognitive effects of acute phenylalanine and tyrosine depletion in patients recovered from depression. Neuropsychopharmacology.

[31] Yin B., Cai Y., Teng T., Wang X., Liu X., Li X., Wang J., Wu H., He Y., Ren F. (2024). Identifying plasma metabolic characteristics of major depressive disorder, bipolar disorder, and schizophrenia in adolescents. Transl. Psychiatry.

[32] Gomez-Merino D., Bequet F., Berthelot M., Riverain S., Chennaoui M., Guezennec C.Y. (2001). Evidence that the branched-chain amino acid L-valine prevents exercise-induced release of 5-HT in rat hippocampus. Int. J. Sports Med..

[33] Beneyto M., Kristiansen L.V., Oni-Orisan A., McCullumsmith R.E., Meador-Woodruff J.H. (2007). Abnormal glutamate receptor expression in the medial temporal lobe in schizophrenia and mood disorders. Neuropsychopharmacology.

[34] Kruse A.O., Bustillo J.R. (2022). Glutamatergic dysfunction in Schizophrenia. Transl. Psychiatry.

[35] McCutcheon R.A., Krystal J.H., Howes O.D. (2020). Dopamine and glutamate in schizophrenia: Biology, symptoms and treatment. World Psychiatry.

[36] Zarate C., Machado-Vieira R., Henter I., Ibrahim L., Diazgranados N., Salvadore G. (2010). Glutamatergic modulators: The future of treating mood disorders?. Harv. Rev. Psychiatry.

[37] Bernstein H.G., Keilhoff G., Laube G., Dobrowolny H., Steiner J. (2021). Polyamines and polyamine-metabolizing enzymes in schizophrenia: Current knowledge and concepts of therapy. World J. Psychiatry.

[38] Spathopoulou A., Sauerwein G.A., Marteau V., Podlesnic M., Lindlbauer T., Kipura T., Hotze M., Gabassi E., Kruszewski K., Koskuvi M. (2024). Integrative metabolomics-genomics analysis identifies key networks in a stem cell-based model of schizophrenia. Mol. Psychiatry.

[39] Guo L., Zhang T., Li R., Cui Z.Q., Du J., Yang J.B., Xue F., Chen Y.H., Tan Q.R., Peng Z.W. (2022). Alterations in the Plasma Lipidome of Adult Women With Bipolar Disorder: A Mass Spectrometry-Based Lipidomics Research. Front. Psychiatry.

[40] Li M., Gao Y., Wang D., Hu X., Jiang J., Qing Y., Yang X., Cui G., Wang P., Zhang J. (2022). Impaired Membrane Lipid Homeostasis in Schizophrenia. Schizophr. Bull..

[41] Rege S., Mackworth-Young C. (2015). Antiphospholipid antibodies as biomarkers in psychiatry: Review of psychiatric manifestations in antiphospholipid syndrome. Transl. Dev. Psychiatry.

[42] Matam Y., Ray B.D., Petrache H.I. (2016). Direct affinity of dopamine to lipid membranes investigated by Nuclear Magnetic Resonance spectroscopy. Neurosci. Lett..

[43] Zhuo C., Hou W., Tian H., Wang L., Li R. (2020). Lipidomics of the brain, retina, and biofluids: From the biological landscape to potential clinical application in schizophrenia. Transl. Psychiatry.

[44] Xie J., Zhong Q., Wu W.-t., Chen J.-j. (2023). Multi-omics data reveals the important role of glycerophospholipid metabolism in the crosstalk between gut and brain in depression. J. Transl. Med..

[45] Rezin G.T., Amboni G., Zugno A.I., Quevedo J., Streck E.L. (2009). Mitochondrial dysfunction and psychiatric disorders. Neurochem. Res..

[46] Kivimaki M., Shipley M.J., Batty G.D., Hamer M., Akbaraly T.N., Kumari M., Jokela M., Virtanen M., Lowe G.D., Ebmeier K.P. (2014). Long-term inflammation increases risk of common mental disorder: A cohort study. Mol. Psychiatry.

[47] Giesbrecht C.J., O’ Rourke N., Leonova O., Strehlau V., Paquet K., Vila-Rodriguez F., Panenka W.J., MacEwan G.W., Smith G.N., Thornton A.E. (2016). The Positive and Negative Syndrome Scale (PANSS): A Three-Factor Model of Psychopathology in Marginally Housed Persons with Substance Dependence and Psychiatric Illness. PLoS ONE.

[48] Soron T.R. (2017). Validation of Bangla Montgomery Asberg Depression Rating Scale (MADRSB). Asian J. Psychiatry.

[49] Hobson J. (2015). The Montreal Cognitive Assessment (MoCA). Occup. Med..

[50] Licht R.W., Jensen J. (1997). Validation of the Bech-Rafaelsen Mania Scale using latent structure analysis. Acta Psychiatr. Scand..

[51] Ducatez F., Mauhin W., Boullier A., Pilon C., Pereira T., Aubert R., Benveniste O., Marret S., Lidove O., Bekri S. (2021). Parsing Fabry Disease Metabolic Plasticity Using Metabolomics. J. Pers. Med..

[52] Tebani A., Mauhin W., Abily-Donval L., Lesueur C., Berger M.G., Nadjar Y., Berger J., Benveniste O., Lamari F., Laforêt P. (2020). A Proteomics-Based Analysis Reveals Predictive Biological Patterns in Fabry Disease. J. Clin. Med..

[53] Eriksson L., Trygg J., Wold S. (2014). A chemometrics toolbox based on projections and latent variables. J. Chemom..

[54] Van Den Berg R.A., Hoefsloot H.C., Westerhuis J.A., Smilde A.K., Van Der Werf M.J. (2006). Centering, scaling, and transformations: Improving the biological information content of metabolomics data. BMC Genom..

[55] Ritchie M.E., Phipson B., Wu D., Hu Y., Law C.W., Shi W., Smyth G.K. (2015). limma powers differential expression analyses for RNA-sequencing and microarray studies. Nucleic Acids Res..

[56] Benjamini Y., Hochberg Y. (1995). Controlling the false discovery rate: A practical and powerful approach to multiple testing. J. R. Stat. Soc. Ser. B.

[57] Wright M.N., Ziegler A. (2017). ranger: A Fast Implementation of Random Forests for High Dimensional Data in C++ and R. J. Stat. Softw..

[58] Kuhn M. (2020). Caret: Classification and Regression Training. https://CRAN.R-project.org/package=caret.

